# Chronic Obstructive Pulmonary Disease and the Omicron Variant of COVID-19 Prognosis: A Retrospective Cohort Study

**DOI:** 10.7759/cureus.65713

**Published:** 2024-07-29

**Authors:** Cheuk Cheung Derek Leung, Ellen Lok Man Yu, Yu Hong Chan, Man Ying Ho, Chin Tong Kwok, Hiu Ching Christy Chan, Yiu Cheong Yeung

**Affiliations:** 1 Medicine and Geriatrics, Princess Margaret Hospital, Hong Kong, HKG; 2 Clinical Research Centre, Kowloon West Cluster, Hong Kong, HKG; 3 Respiratory Medicine, Kowloon Hospital, Hong Kong, HKG

**Keywords:** retrospective cohort, omicron variant, morbidity and mortality, copd: chronic obstructive pulmonary disease, coronavirus disease 2019 (covid-19)

## Abstract

Background and aim: This retrospective cohort study aimed to investigate the association between chronic obstructive pulmonary disease (COPD) and the prognosis of COVID-19 patients infected with the Omicron variant. The primary objective was to determine if COVID-19 patients with COPD had higher mortality rates compared to those without COPD. Secondary objectives included assessing the risk of respiratory failure, hospital stay length, intensive care unit (ICU) admission, and oxygen requirements in COPD patients with COVID-19.

Materials and methods: The study included 2761 COVID-19 patients admitted to the Princess Margaret Hospital, Hong Kong, between January 1 and June 30, 2022. Among them, 7.4% (n = 205) had COPD. Demographic and clinical data, including vaccination status and comorbidities, were collected. The primary outcome was 30-day mortality, and secondary outcomes included respiratory support requirement, hospital stay length, and ICU admission. Logistic regression analyses were conducted, adjusting for potential confounders.

Results: COPD did not independently increase the risk of COVID-19 mortality after adjusting for confounders. Instead, older age, male sex, incomplete vaccination, long-term oxygen therapy use, and specific comorbidities were identified as significant predictors of 30-day mortality. COPD patients were more likely to require oxygen and noninvasive ventilation, but there were no significant differences in other secondary outcomes compared to non-COPD patients.

Conclusion: COPD itself was not an independent risk factor for COVID-19 mortality. Age, sex, vaccination status, comorbidities, and long-term oxygen therapy use were important predictors of mortality. These findings underscore the importance of considering multiple factors when assessing the impact of COPD on COVID-19 prognosis, particularly with the Omicron variant.

## Introduction

Coronavirus disease 2019 (COVID-19) is caused by severe acute respiratory syndrome coronavirus (SARS-CoV-2) infection. It remains a pandemic with substantial morbidity and mortality [1]. The severity of the disease ranges from asymptomatic infection to severe pneumonia with respiratory failure and death [2]. A new variant of SARS-CoV-2 with lineage B.1.1.529, named Omicron, emerged in November 2021 [3]. Compared to previously circulating variants, it has greater transmissibility while demonstrating lower virulence and decreased mortality [4,5]. The Omicron variant caused the fifth wave of COVID-19 in Hong Kong since December 31, 2021 [6]. As of June 30, 2022, there were more than 1.2 million cases of COVID-19 in Hong Kong, with more than 9,000 deaths recorded [7].

The attachment of SARS-CoV-2 to host cells occurs through the binding of the spike protein to angiotensin-converting enzyme 2 (ACE-2), and transmembrane protease serine 2 (TMPRSS2) plays a role in facilitating viral entry. Variances in the levels of expression of ACE2 and TMPRSS2 could potentially impact an individual's susceptibility to SARS-CoV-2 infection and affect the clinical course of the disease [8,9].

Chronic obstructive pulmonary disease (COPD) is a condition characterized by chronic respiratory symptoms due to underlying airway disease and/or alveoli abnormalities, causing persistent airflow obstruction [10]. These patients have increased expression of ACE-2 receptors in small airways [11]. Some literature reported that COPD independently increases the likelihood of severe COVID-19 illness or mortality [9,12-20], but others did not [21-26]. All these studies examined the population before the emergence of the Omicron variant of SARS-CoV-2. Given conflicting results from various literature and the constant evolution of COVID-19, we conducted a retrospective cohort study to analyze the impact of COPD on COVID-19 severity and mortality in a population predominantly infected with the Omicron variant. To our knowledge, this relationship has not been studied in the Hong Kong population before.

Objectives

This study aims to evaluate the impact of COPD on COVID-19 prognosis. We hypothesized that compared to COVID-19 patients without COPD, those with COPD have higher chances of respiratory failure and a higher mortality rate.

The primary objective of this study was to determine if COPD patients infected with COVID-19 had a higher mortality rate than those without COPD. The secondary objectives were to determine if COPD patients infected with COVID-19 were more likely to require oxygen, longer length of hospitalization, intensive care unit (ICU) admission, and develop respiratory failure than those without COPD and to identify the independent risk factors for COVID-19 mortality with multivariate analysis.

## Materials and methods

Study design

This is a retrospective cohort study of COVID-19 patients admitted to the Princess Margaret Hospital (PMH). PMH is a tertiary hospital in Hong Kong providing 24-hour emergency service in the Kowloon West Cluster. It is also home to the Hospital Authority (HA) Infectious Disease Centre, the largest isolation inpatient facility in Hong Kong.

A list of patients admitted to PMH between January 1, 2022, and June 30, 2022, with a diagnosis of COVID-19 was generated from the HA Clinical Data Analysis and Reporting System (CDARS). They were categorized into the COPD and non-COPD groups. Data including age, sex, COVID-19 vaccination status, place of residency, smoking history, COPD medications, long-term oxygen (LTOT) use, medical comorbidities, and COVID-19 treatment were collected by reviewing their electronic and written patient records.

Inclusion and exclusion criteria

Subjects included in this study were adult patients who were at least 40 years old and admitted to PMH between January 1, 2022, and June 30, 2022, diagnosed with COVID-19 as evidenced by the International Classification of Diseases, Ninth Revision, Clinical Modification (ICD-9-CM) code of 519.8 (8) and a positive nucleic acid real-time polymerase chain reaction (RT-PCR) test for severe acute respiratory syndrome coronavirus 2 (SARS-CoV-2). Those younger than 40 years or without a positive SARS-CoV-2 RNA RT-PCR test were excluded.

Outcomes

The primary outcome measured was 30-day mortality. Secondary outcomes included oxygen use, need for a non-rebreather oxygen mask, high-flow nasal cannula, invasive or noninvasive mechanical ventilation, extracorporeal membrane oxygenation (ECMO), length of hospital stay, and ICU admission.

Definitions

COPD was defined when all these three criteria were met: documented ICD-9-CM code of COPD including 490, 491, 492, and 496; documented self-reported history of smoking, and outpatient COPD medication use. Patients with documented coexisting pulmonary diseases, including interstitial lung disease, bronchiectasis, and asthma-COPD overlap syndrome, were excluded from the COPD group. Spirometry was not considered in defining COPD as most patients did not have one performed recently.

Completed COVID-19 vaccination, as of June 30, 2022, was defined as two or more doses of COVID-19 vaccines recognized by the Hong Kong government [27]. Most patients with completed COVID-19 vaccination either received two or more doses of CoronaVac or BNT162b2, which were freely available to the residents of Hong Kong.

The COPD medications included short-acting beta-2 agonists (SABA); short-acting muscarinic antagonists (SAMA); long-acting beta-2 agonists (LABA); long-acting muscarinic antagonists (LAMA); inhaled corticosteroid (ICS); combinations of LABA/LAMA, ICS/LABA, and ICS/LABA/LAMA; phosphodiesterase-4 (PDE-4) inhibitors; methylxanthines, and systemic beta-agonists.

The Charlson comorbidity index (CCI) predicts the mortality of patients with multiple comorbidities [28]. Data of comorbidities listed in CCI, including myocardial infarction, congestive heart failure, peripheral vascular disease (PVD), cerebrovascular disease, dementia, chronic pulmonary disease, rheumatologic disease, peptic ulcer disease, liver disease, diabetes mellitus, renal disease, solid tumors, leukemia, lymphoma, and human immunodeficiency virus (HIV) infection, were recorded by reviewing electronic and written patient records.

The length of hospital stay was calculated by subtracting the date of admission from the date of discharge. If the patient was transferred to another hospital under HA, it would still be counted as the same admission. If the patient was discharged on the first day of admission, the length of hospitalization was counted as one day.

Respiratory failure was defined as the use of a non-rebreather oxygen mask, high-flow nasal cannula, invasive or noninvasive mechanical ventilation, or ECMO. PaO_2_ measurement was not considered in defining respiratory failure as some of the patients did not have an arterial blood gas test.

Oxygen use was counted if oxygen was given at any point of the patient's COVID-19 admission, even for LTOT users without increment of their baseline oxygen.

Care service residency included patients who lived in residential care homes for the elderly, residential care homes for the disabled, and hostels for severely and moderately mentally handicapped persons, before their COVID-19 admission.

Statistics

COVID-19 patients in the COPD and non-COPD groups were compared using the Fisher's exact test or Chi-square test of independence for categorical variables where appropriate. The Mann-Whitney U-test was used for continuous variables. The same statistical analysis was applied to the subgroup analysis between ICS and non-ICS groups. Univariate and multivariate analyses were conducted using logistic regression to identify risk factors independently associated with mortality. For the multivariate analyses, the variance inflation factor for each independent variable was checked for multicollinearity. Risk factors were reported as adjusted odds ratios (OR_adj_) with 95% confidence intervals (CI). A P-value < 0.05 was considered statistically significant. All statistical analyses were conducted by SPSS software, version 29 (IBM Corp., Armonk, NY).

## Results

**Figure 1:**
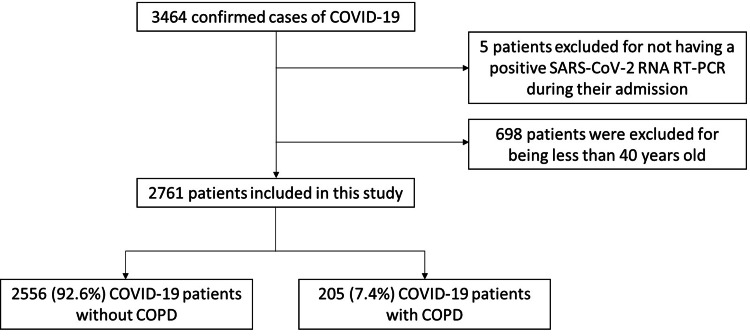
Flowchart of the inclusion and exclusion of subjects in the study SARS-CoV-2: Severe acute respiratory syndrome coronavirus; RT-PCR: Real-time polymerase chain reaction; COPD: Chronic obstructive pulmonary disease.

Among 3464 patients admitted to PMH between January 1, 2022, and June 30, 2022, diagnosed with COVID-19 with an ICD-9-CM code of 519.8 (8), five patients were excluded for not having a positive SARS-CoV-2 RNA RT-PCR during their admission. Another 698 patients were excluded because they were younger than 40 years. The remaining 2761 patients were included, and 205 (7.4%) and 2556 (92.6%) patients were categorized into the COPD and non-COPD groups, respectively (Figure 1).

Patient characteristics

Table 1 shows the baseline characteristics of the study population. COPD patients were significantly older (mean age = 81.9 vs 77.8 years, P < 0.001) and had a higher proportion of male sex (90.7% vs 49.1%, P < 0.001). All COPD patients had a smoking history (as one of their inclusion criteria) when compared to 27.3% of patients in the non-COPD group (P < 0.001). The proportion of current smokers also differed significantly between the two groups (23.4% for COPD vs 5.1% for non-COPD, P < 0.001). COPD patients were also more commonly on long-term oxygen (17.1% vs 0.9%, P < 0.001).

**Table 1:** Patient characteristics in non-COPD versus COPD groups ^a^ Mann–Whitney U-test. ^b^ Chi-square test of independence. ^c^ Fisher's exact test. *Statistically significant results (P-value ≤ 0.05). SD: Standard deviation; COPD: Chronic obstructive pulmonary disease; HIV: Human immunodeficiency virus; AIDS: Acquired immune deficiency syndrome.

	Non-COPD (n = 2556)	COPD (n = 205)	P-value
Age, mean (SD)^a^	77.8 (13.46)	81.9 (8.6)	<0.001*
Sex, male, n (%)^b^	1257 (49.1%)	186 (90.7%)	<0.001*
Residency, n (%)^b^			0.027*
Home, n (%)^b^	1481 (57.9%)	135 (65.9%)	
Care service residency, n (%)^b^	1075 (42.1%)	70 (34.1%)	
Vaccination completion, n (%)^b^	718 (28.1%)	44 (21.5%)	0.041*
Ex-smoker or current smoker, n (%)^b^	698 (27.3%)	205 (100%)	<0.001*
Current smoker, n (%)^b^	131 (5.1%)	48 (23.4%)	<0.001*
Long-term oxygen, n (%)^b^	22 (0.9%)	35 (17.1%)	<0.001*
Comorbidities, n (%)			
Myocardial infarction^b^	180 (7%)	13 (6.3%)	0.705
Heart failure^b^	230 (9.0%)	21 (10.2%)	0.551
Peripheral vascular disease^c^	54 (2.1%)	7 (3.4%)	0.214
Cerebrovascular disease^b^	586 (22.9%)	50 (24.4%)	0.632
Dementia^b^	471 (18.4%)	24 (11.7%)	0.016*
Chronic lung disease^b^	131 (5.1%)	205 (100%)	<0.001*
Connective tissue disease^c^	59 (2.3%)	5 (2.4%)	0.810
Ulcer disease^b^	110 (4.3%)	16 (7.8%)	0.021*
Liver disease^b^	74 (2.9%)	4 (2.0%)	0.433
Diabetes mellitus^b^	881 (34%)	30 (14.5%)	<0.001*
Moderate to severe renal disease^b^	189 (7.4%)	3 (1.5%)	0.001*
Solid tumor^b^	331 (12.9%)	35 (17.1%)	0.094
Lymphoma or leukemia^c^	47 (1.8%)	4 (2.0%)	0.789
HIV/AIDS^c^	4 (0.2%)	0 (0%)	1.000
Charlson comorbidity index, median (SD)^a^	5.03 (2.22)	6.19 (2.01)	<0.001*

The non-COPD group has a larger proportion of residents of care services when compared to the COPD group (42.1% vs 34.1%, P = 0.027). More patients have completed COVID-19 vaccination in the non-COPD group too (28.1% vs 21.5%, P = 0.041).

Most comorbidities were comparable between the two groups, apart from chronic lung disease and ulcer disease, which were more frequently present in the COPD group. Dementia, diabetes mellitus, and renal disease were also more prevalent in the non-COPD group. CCI was significantly higher in the COPD group (mean = 6.19) when compared to the non-COPD group (mean = 5.03, P < 0.001).

Univariate logistic regression found an OR of 1.24 (95% CI: 1.19-1.29, P < 0.001) showing a positive association between a higher CCI and incomplete vaccination. Furthermore, it also found an OR of 1.04 (95% CI: 1.03-1.04, P < 0.001) indicating a correlation between older age and incomplete vaccination.

Treatment and clinical outcomes

Table 2 shows the treatment received and clinical outcomes of the study population. The COVID-19 treatment received, including antibiotics, antivirals (remdesevir, molnupiravir, or Paxlovid), interferon, baricitinib, tocilizumab, heparin (unfractionated or low-molecular-weight heparin), and vasopressors did not differ between the two groups. A significantly higher proportion of patients received corticosteroids (82% vs 41.6%, P < 0.001) in the COPD group when compared with the non-COPD group.

**Table 2:** Treatment and outcomes in non-COPD versus COPD groups ^a^ Mann–Whitney U-test. ^b^ Chi-square test of independence. ^c^ Fisher's exact test. *Statistically significant results (P-value ≤ 0.05). COPD: Chronic obstructive pulmonary disease; ECMO: Extracorporeal membrane oxygenation; ICU: Intensive care unit; IQR: Interquartile range; LMWH: Low-molecular-weight heparin.

	Non-COPD (n = 2556)	COPD (n = 205)	P-value
Antivirals (remdesivir, molnupiravir or Paxlovid), n (%)^b^	623 (24.4%)	57 (27.8%)	0.273
Remdesivir, n (%)^b^	162 (6.3%)	19 (9.3%)	0.103
Molnupiravir, n (%)^b^	272 (10.6%)	30 (14.6%)	0.078
Paxlovid, n (%)^b^	202 (7.9%)	10 (4.9%)	0.118
Interferon, n (%)^c^	31 (1.2%)	4 (2.0%)	0.326
Steroid, n (%)^b^	1063 (41.6%)	168 (82%)	<0.001*
Baricitinib, n (%)^c^	45 (1.8%)	1 (0.5%)	0.254
Tocilizumab, n (%)^c^	20 (0.8%)	1 (0.5%)	1.000
LMWH/heparin, n (%)^b^	583 (22.8%)	58 (28.3%)	0.074
Vasopressor, n (%)^b^	116 (4.5%)	12 (5.9%)	0.389
Clinical outcomes			
Oxygen use, n (%)^b^	1280 (50.1%)	175 (85.4%)	<0.001*
Respiratory failure, n (%)^b^	746 (29.2%)	72 (35.1%)	0.073
Non-rebreather oxygen mask, n (%)^b^	621 (24.3%)	55 (26.8%)	0.417
High-flow nasal cannula, n (%)^c^	44 (1.7%)	6 (2.9%)	0.266
Noninvasive ventilation, n (%)^c^	4 (0.2%)	3 (1.5%)	0.011*
Mechanical ventilation, n (%)^b^	83 (3.2%)	8 (3.9%)	0.613
ECMO^c^	1 (0.04%)	0	1.000
ICU admission, n (%)^b^	82 (3.2%)	4 (2.0%)	0.319
30-day mortality, n (%)^b^	639 (25%)	64 (31.2%)	0.049*
Length of stay (days), median (IQR)^a^	9 [4,15]	8 [4,15]	0.384

Our primary outcome, 30-day mortality, was significantly higher in the COPD group (31.2% vs 25%, P = 0.049). As for secondary outcomes, the development of respiratory failure, length of hospital stay, and number of ICU admissions did not differ between the two groups. The proportion of patients who required oxygen (85.4% vs 50.1%, P < 0.001) and noninvasive ventilation (NIV) (1.5% vs 0.2%, P = 0.011) was significantly higher in the COPD group. There was no difference in the use of a non-rebreather oxygen mask, high-flow nasal cannula, or invasive mechanical ventilation. Among the study population, only one non-COPD patient required ECMO, and the difference did not reach statistical significance between the two groups.

A higher percentage of patients were treated with steroids in the COPD group than the non-COPD group. A similar pattern is also found with a higher percentage of patients in the COPD group requiring oxygen. In the non-COPD group, 41.6% received steroid treatment, while 50.1% required oxygen. In the COPD group, 82% received steroid treatment, while 85.4% required oxygen. Given the significant resemblance in the usage of oxygen and steroids, a logistic regression analysis was conducted to evaluate the relationship between steroid and oxygen use in the COPD group, revealing an odds ratio (OR) of 31.51 (95% CI: 25.22-39.37, P < 0.001).

Risk factors for 30-day mortality in COVID-19 patients

Table 3 shows the univariate and multivariate analyses of risk factors for 30-day mortality in COVID-19 patients. Preliminary unadjusted analysis using univariate logistic regression showed that COPD was associated with higher 30-day mortality, with unadjusted OR (OR_unadj_) = 1.36 (P = 0.05). Other variables associated with 30-day mortality included older age, male sex, care service residency, incomplete vaccination, LTOT use, myocardial infarction, heart failure, PVD, cerebrovascular disease, dementia, renal disease, solid tumors, and higher CCI.

**Table 3:** Univariate and multivariate logistic regression analyses of risk factors associated with 30-day mortality Multivariate analysis was conducted with statistically significant risk factors in univariate analysis. * Statistically significant results (p-value ≤ 0.05). COPD: Chronic obstructive pulmonary disease; CI: Confidence interval; HIV: Human immunodeficiency virus; AIDS: Acquired immune deficiency syndrome.

	Univariate		Multivariate	
	OR_unadj_ (95% CI)	P-value	OR_adj_ (95% CI)	P-value
COPD	1.36 (1.00-1.85)	0.050*	0.88 (0.62-1.26)	0.486
Age	1.04 (1.03-1.04)	<0.001*	1.03 (1.02-1.04)	<0.001*
Sex, male	1.40 (1.15-1.69)	<0.001*	1.58 (1.31-1.92)	<0.001*
Care service residency	2.12 (1.78-2.52)	<0.001*	1.72 (1.42-2.10)	<0.001*
Incomplete vaccination	3.07 (2.43-3.88)	<0.001*	2.28 (1.78-2.92)	<0.001*
Smoking history	0.91 (0.56-1.46)	0.689		
Current smoker	0.83 (0.58-1.20)	0.323		
Long-term oxygen	2.34 (1.37-3.97)	0.002*	2.23 (1.24-4.04)	0.008*
Comorbidities				
Myocardial infarction	1.39 (1.01-1.90)	0.043*	1.27 (0.90-1.80)	0.175
Heart failure	1.33 (1.00-1.77)	0.047*	0.96 (0.70-1.31)	0.782
Peripheral vascular disease	3.12 (1.87-5.19)	<0.001*	2.00 (1.16-3.44)	0.013*
Cerebrovascular disease	1.48 (1.22-1.80)	<0.001*	1.01 (0.81-1.27)	0.916
Dementia	1.64 (1.33-2.21)	<0.001*	1.05 (0.82-1.34)	0.727
Chronic lung disease	1.12 (0.87-1.45)	0.389		
Connective tissue disease	1.15 (0.66 – 2.00)	0.621		
Ulcer disease	1.18 (0.79-1.75)	0.413		
Liver disease	1.08 (0.65-1.80)	0.764		
Diabetes mellitus	1.07 (0.89-1.28)	0.455		
Moderate to severe renal disease	1.71 (1.26-2.33)	<0.001*	1.94 (1.32-2.84)	0.001*
Solid tumor	1.61 (1.27-2.03)	<0.001*	1.55 (1.08-2.23)	0.017*
Lymphoma or leukemia	0.80 (0.41-1.57)	0.520		
HIV/AIDS	0.98 (0.10-9.40)	0.983		
Charlson comorbidity index	1.24 (1.19-1.29)	<0.001*	1.07 (1.00-1.15)	0.048*

With these covariates identified by the univariate analysis, the OR_adj_ was calculated using multivariate logistic regression. COPD was not an independent risk factor for COVID-19 mortality (OR_adj_ = 0.88, P = 0.486). The risk factors independently associated with the COVID-19 30-day mortality were older age (OR_adj_ = 1.03), male sex (OR_adj_ = 1.58), care service residency (OR_adj_ = 1.72), incomplete vaccination (OR_adj_ = 2.28), LTOT use (OR_adj_ = 2.23), PVD (OR_adj_ = 2.00), moderate to severe renal disease (OR_adj_ = 1.94), solid tumors (OR_adj_ = 1.55), and higher CCI (OR_adj_ = 1.07).

## Discussion

This is a rare study to assess the impact of COPD on the prognosis and clinical characteristics of COVID-19 patients in Hong Kong. It is also the first study on such a topic on a population infected with the Omicron variant of SARS-CoV-2.

Our primary outcome, 30-day mortality, was significantly higher in the COPD group (31.2% vs 25%, P = 0.049). However, after adjusting with variables associated with 30-day mortality including older age, male sex, care service residency, incomplete vaccination, LTOT use, myocardial infarction, heart failure, PVD, cerebrovascular disease, dementia, renal disease, solid tumors, and higher CCI, COPD itself was found to be not an independent risk factor for COVID-19 30-day mortality (OR_adj_ = 0.88, 95% CI: 0.62-1.26, P = 0.486).

This interesting statistical result was directly influenced by the difference in baseline characteristics between the non-COPD and COPD cohorts. Our COPD patients were significantly older, predominantly male with a lower COVID-19 vaccination rate. These were the variables associated with higher 30-day mortality. Some literature reported that COPD independently increases the likelihood of severe COVID-19 illness or mortality [9,12-20], but others did not [21-26]. The SARS-CoV-2 virus demonstrates a distinct preference for affecting the lungs, resulting in the development of COVID-19 pneumonia. Considering the inflammation and impairment of gas exchange associated with COVID-19, it is logical to assume that individuals with pre-existing pulmonary conditions are at an increased risk for experiencing a more severe disease progression and a deteriorating clinical trajectory. This study contributes a supplementary and more equitable perspective to the predominantly held position of COPD representing an absolute risk factor associated with unfavorable outcomes in the context of COVID-19.

The use of CCI to predict mortality in COPD patients has been well validated [29,30]. Therefore, it was chosen over other scoring systems, and its individual components were compared between the COPD and non-COPD groups. COPD patients are more likely to have pre-existing comorbidities including diabetes mellitus, cardiovascular diseases, and stroke [31]; however, our COPD group had a lower prevalence of diabetes and a similar prevalence of cardiovascular disease and stroke compared to the non-COPD group. Even though the median CCI was higher in the COPD group, it is reasonable to assume that our non-COPD cohort has significantly more comorbidities than the general population, possibly accounting for the higher 30-day mortality before adjustment. The non-COPD group was also frailer than the public, reflected by a higher proportion of care service residents than the COPD group. This selection bias was a result of hospital bed shortage during the COVID-19 surge, which peaked in March 2022, when only patients with more severe disease and worse outcomes were admitted to hospitals.

In terms of treatment received, only corticosteroid was prescribed more often in the COPD group. All other medications including antibiotics, antivirals, anticoagulants, and immunomodulatory drugs were comparable between the two groups. This difference could be explained by two reasons. First, the recovery trial showed that among patients admitted to the hospital with COVID-19, the administration of dexamethasone (intravenous or oral, 6 mg daily, up to 10 days) led to reduced mortality rates at 28 days for individuals who were receiving invasive mechanical ventilation or oxygen alone [32]. This practice was incorporated into the local treatment guideline [33]. Patients of the COPD group were more likely to require oxygen, which led to a higher prescription of corticosteroids (OR = 31.51 95% CI: 25.22-39.37, P < 0.001)). Second, corticosteroid (oral prednisolone or intravenous hydrocortisone) is also commonly prescribed for acute exacerbation of COPD (AECOPD), which may be triggered by the COVID-19 infection [34].

One of the significant findings in our study was a higher proportion of patients required oxygen (85.4% vs 50.1%) and NIV (1.5% vs 0.2%) in the COPD group compared to the non-COPD group. The increased use of oxygen was in keeping with results from a similar study [21], but it was not listed as an outcome measured in other kinds of literature [9,12-20,22-26]. It is important to note that although oxygen was used more often in the COPD group, there was no difference in the prevalence of respiratory failure (defined as the use of non-rebreather oxygen mask, high-flow nasal cannula, invasive or noninvasive mechanical ventilation, or ECMO) between the two groups. Similarly, NIV itself was not listed as an outcome variable in previous studies [9,12-26]. In Hong Kong, before COVID-19, NIV was used in 18.4% of the headcount of inpatients admitted for COPD in the year 2014, according to a local study [35]. During the COVID-19 pandemic, NIV was only used in 1.5% of COPD patients in our study (Table 2), which was remarkably lower than the 2014 rates. This decline can be attributed to the prior knowledge gained from the severe acute respiratory syndrome (SARS) outbreak in 2003. It was observed that NIV posed a risk to healthcare workers due to the potential transmission of the virus through aerosols containing viral particles [35,36].

Factors independently increasing the risk of COVID-19 30-day mortality included older age, male sex, care service residency, incomplete vaccination, LTOT use, and comorbidities including PVD, moderate to severe renal disease, solid tumors, and higher CCI. It comes as no surprise that older patients are more likely to die of COVID. However, male sex, regardless of age, was a risk factor for COVID-19 mortality. A meta-analysis quoted men had a relative risk of mortality of 1.36 (95% CI: 1.17-1.59, P < 0.01) compared with women. The exact cause of this phenomenon is unknown, but the study suggested it may be related to testosterone having effects on disease severity or health behaviors such as men being more prone to alcohol and tobacco use [37].

The OR_adj_ of 30-day mortality of care service residents was 1.72 (95% CI: 1.42-2.10, P < 0.001). In comparison with those living in the community, care service residents are older and frailer with multiple comorbidities [38] and are expected to have poorer outcomes with COVID-19. According to an observational study conducted in Hong Kong, COVID-19 mortality rates exhibited a progressive increase as frailty levels escalated. The study found that mortality ranged from 5.7% in patients who were considered fit and well to 40% in patients with at least severe frailty [39]. A study in the United States reported that higher-quality care homes have lower COVID-19 mortality [40]. Hence, the substandard quality of care homes in Hong Kong, characterized by inadequately designed indoor environments, insufficient ventilation, cramped spaces, and a lack of resilience to COVID-19 outbreaks, emerges as another potential factor contributing to higher mortality rates among care service residents [41].

Our study identified incomplete COVID-19 vaccination (fewer than two doses), with OR_adj_ of 2.28 (95% CI: 1.78-2.92, P < 0.001), as a risk factor for mortality. The two COVID-19 vaccines made available to Hong Kong's public were CoronaVac and BNT162b2. A population-based observational study reported that within 28 days of a positive COVID-19 test, the administration of two doses of CoronaVac or BNT162b2 protected against severe illness and death. Among adults aged 60 years or older, BNT162b2 and CoronaVac exhibited effectiveness of 89.3% (95% CI: 86.6-91.6) and 69.9% (95% CI: 64.4-74.6), respectively, against COVID-19 [42]. Another study quoted that the risk of COVID-19 death in adults aged 60 years or older was 20 times lower among those who were fully vaccinated compared with those who were unvaccinated [43]. These results are in keeping with our study's findings.

The vaccination rate was very low among the study population, with only 28.1% and 21.5% having completed vaccination in the non-COPD and COPD cohorts, respectively. A retrospective study found that individuals belonging to the older age groups (70 years and above, particularly those aged 80 years and above), living in urban areas, having functional dependence, and having chronic conditions, exhibited a lower likelihood of receiving COVID-19 vaccines [44]. These factors coincided with our study population. The correlation between higher CCI and incomplete vaccination was confirmed by logistic regression analysis, revealing an OR of 1.24 (95% CI: 1.19-1.29, P < 0.001). On the other hand, older age was also correlated with incomplete vaccination (OR = 1.04, 95% CI: 1.03-1.04, P < 0.001). The COPD group was older with higher CCI, which could explain their lower vaccination rate compared to the non-COPD group (21.5% vs 28.1%, P = 0.041).

Our study identified higher CCI and comorbidities, including PVD, moderate to severe renal disease, and solid tumors, as factors independently linked to a higher risk of 30-day COVID-19 mortality. On review of prior literature, PVD was reported to be independently associated with increased COVID-19 mortality, with an OR of 1.45 (95% CI: 1.11-1.88) [45]. History of end-stage renal failure or dialysis was also associated with increased COVID-19 mortality risk (hazard ratio: 3.69, 95% CI: 3.09-4.39) [46], and a meta-analysis reported that patients with pre-diagnosed cancer were more likely to die based on 96 articles with 6,518,992 COVID-19 patients (pooled OR = 1.47, CI: 1.31-1.65) [47]. These findings align with the results of our study.

Univariate analysis in our study identified that components of CCI, including myocardial infarction, heart failure, cerebrovascular disease, and dementia, increased COVID-19 mortality risk. However, after adjusting for age, sex, care service residency, incomplete vaccination, LTOT use, and CCI, this correlation no longer reached statistical significance. Other CCI components including connective tissue disease, ulcer disease, liver disease, and diabetes mellitus were also associated with increased 30-day mortality (OR_unadj_ > 1) but did not reach statistical significance. Lymphoma or leukemia and HIV were associated with decreased COVID-19 30-day mortality (OR_unadj_ < 1) without statistical significance, and their sample size was small (n = 51 and n = 4, respectively). All these comorbidities have been reported to increase COVID-19 mortality in meta-analysis or nationwide studies with a large sample size [46,48]. A relatively small sample size (n = 2761) is perhaps the answer to the discrepancy between our study and prior literature.

Although the exact prevalence of COPD in Hong Kong is unknown, it was estimated to be 9% among the elderly aged 70 or above [49] by one study and 12.4%-25.9% based on another study in the population aged 60 or above [50]. Only 7.4% of our study population had COPD, which was a lower percentage than expected. However, they have a high mortality of more than 30%, which was much higher than the mortality of 20% in COPD patients infected with the Omicron variant reported by an Indian study [5]. Throughout the six-month study period, COVID-19 admissions reached their peak, while there were only 343 admissions for AECOPD without COVID-19. This represents a substantial decrease compared to the pre-pandemic period, specifically in 2019 when there were 1156 admissions for AECOPD to PMH during the same time frame according to data retrieved from CDARS. Studies have demonstrated a reduction in AECOPD admissions both locally [51] and worldwide [52,53]. COPD patients were speculated to avoid seeking medical attention during exacerbation to prevent hospital-acquired infections with COVID-19 [54]. Better adherence to their preventative inhalers and shielding advice have also been reported [55]. Due to bed shortage, instead of being admitted to hospitals, COPD patients with mild COVID-19 symptoms were attended to in the designated clinics established by the Hong Kong government, effectively addressing their medical necessities [6]. This leaves only the more severe COPD cases, with or without COVID-19, with worse outcomes to be admitted, possibly explaining the low prevalence of COPD in our study with a high mortality rate.

Several strengths of our study should be highlighted, including its up-to-date nature. A literature search on PubMed, Crossref, and Google Scholar at the time of writing did not identify any studies on the impact of COPD on COVID-19 prognosis with data collected specifically after the emergence of the Omicron variant. Although not verified case by case, the vast majority of our study population was infected with the Omicron variant [56]. As the current circulating SARS-CoV-2 at the time of writing are still sub-lineages of the Omicron variant [57], our study provides more up-to-date information on the interplay between COVID-19 and COPD than the older literature available.

Another notable strength is the large COPD cohort population. In comparison with several other retrospective studies focused on the impact of COPD on COVID-19 outcomes [12,13,21,22], where their COPD sample size ranges from 35 to 141, our study has a notably larger COPD cohort of 205 subjects.

Limitations

There are several limitations to our research that should be stressed. First, it is a retrospective study, which is prone to biases, and the overall findings may result from unmeasured confounding factors. For example, the government's changes in isolation and hospitalization policies during the study period likely had an impact on the length of hospitalization. Additionally, data on past medical history, drug treatment, oxygen use, and the development of respiratory failure might not be accurately documented in the hospital's electronic and written records, introducing potential inaccuracies.

Second, we did not include COPD symptom scores or lung function tests, which are variables used for diagnosing COPD, staging [58], and monitoring disease progression. COPD symptom scores were not well-documented in the clinical notes or discharge summaries. Most COPD patients did not have a recent lung function test, partly due to the COVID-19 outbreak itself, which led to non-essential services being halted in many hospitals in Hong Kong due to the increased strain on inpatient care with many COVID-19 patients. Moreover, it was unclear whether lung function tests should be considered aerosol-generating [59], which further contributed to service suspension and delays to prevent the spread of COVID-19. Since our diagnosis of COPD was based on ICD-9-CM coding, the diagnosis was likely underreported. We added two additional criteria for COPD diagnosis, including self-reported smoking history and outpatient COPD medication use, to improve diagnostic accuracy. However, adding these criteria may have incorrectly classified COPD patients into the "non-COPD" cohort, including COPD patients who were non-smokers.

Third, respiratory failure is usually defined as PaO_2_ < 60 mmHg (8kPa) and is further divided into type I and type II depending on the PaCO_2_. It is one of the secondary outcomes measured in this study, but PaO_2_ measurement was not always available if patients did not have arterial blood gas obtained for analysis. Therefore, respiratory failure in our study was defined as the use of a non-rebreather oxygen mask, high-flow nasal cannula, invasive or noninvasive mechanical ventilation, or ECMO. This definition may have underestimated the number of respiratory failures, as some patients may have had PaO_2_ < 60mmHg but only required oxygen via nasal cannula. Oxygen use itself was not classified as respiratory failure, as inpatient oxygen use was relatively liberal in our locality, which also correlated with increased inpatient steroid use.

Lastly, while the treatment of COVID-19 in the study population was guided by recommendations from the Hospital Authority Central Committee on Infectious Diseases and Emergency Response, the recommendations themselves were updated several times during the study period. Only around 25% of the study population received antiviral treatment. Remdesivir, which has been associated with a 17% lower risk of inpatient mortality among patients hospitalized with COVID-19 [60], was available throughout the study period. The most significant update was the introduction of oral antiviral drugs, such as molnupiravir and Paxlovid, which were available in Hong Kong on March 2, 2022, and March 11, 2022, respectively [61]. Patients treated with molnupiravir or Paxlovid had a lower 30-day risk of death compared with matched, untreated control participants (risk difference: -10.42 (95% CI: -13.49 to -7.35) per 1,000 participants and -4.22 (95% CI: -5.45 to -3.00) per 1,000 participants, respectively) [62]. Changes in guidance made it impossible to standardize treatment across study groups, potentially introducing confounding elements that we were unable to account for.

## Conclusions

In conclusion, after adjusting for factors that independently increase the risk of COVID-19 30-day mortality, COPD was not associated with increased 30-day mortality in patients admitted to a major medical unit infected with COVID-19. Although COPD patients were more likely to require oxygen and NIV, there were no differences in other secondary outcomes, including the need for a non-rebreather oxygen mask, high-flow nasal cannula, invasive mechanical ventilation, ECMO, length of hospital stay, and ICU admission. Factors independently increasing the risk of COVID-19 30-day mortality included older age, male sex, care service residency, incomplete vaccination, LTOT use, and comorbidities including PVD, moderate to severe renal disease, solid tumors, and higher CCI.

Currently, in most regions across the globe, the once-devastating impact of COVID-19 appears to have receded into the annals of history, ushering in a return to the cherished state of pre-pandemic normalcy. However, SARS-CoV-2 is unlikely to disappear completely, and it is inevitable that COVID-19 will eventually transform into an endemic illness. As new variants and viral mutations continue to emerge, it becomes increasingly crucial to deepen our understanding of COVID-19 and its intricate interplay with human illnesses. Studies found that 30% of COVID-19 survivors reported persistent symptoms, and the prevalence was even higher in patients who required hospitalization. Future research may delve into the lasting effects of COVID-19 on COPD patients, shedding light on their long-term consequences.
